# Velvet antler polypeptide partially rescue facet joint osteoarthritis-like phenotype in adult β-catenin conditional activation mice

**DOI:** 10.1186/s12906-019-2607-4

**Published:** 2019-07-30

**Authors:** Wan-qing Xie, Yong-jian Zhao, Feng Li, Bing Shu, Shu-ru Lin, Li Sun, Yong-jun Wang, Hong-xin Zheng

**Affiliations:** 1 Department of Spine Surgery, Peking University Shenzhen Hospital, Shenzhen Peking University-The Hong Kong University of Science and Technology Medical Center, Shenzhen, China; 2 Molecular Laboratory of TCM, Department of Basic Medicine, Liaoning University of TCM, 79 Chongshan East Road, Shenyang, 110032 China; 3 Longhua Hospital, Shanghai University of Traditional Chinese Medicine, 725 South WanPing Road, Shanghai, 200032 China

**Keywords:** Velvet antler polypeptide, Wnt/β-catenin signaling pathway, Facet joint osteoarthritis

## Abstract

**Background:**

Wnt/β-catenin signaling pathway is closely related to osteoarthritis. In our preliminary study, β-catenin conditional activation (cAct) mice that specifically over-express β-catenin gene in cartilage chondrocyte exhibits osteoarthritis-like phenotype in the lumbar disc and knee joint. Therefore, we used the mice to model FJ-OA and test the potential curative effect of Velvet Antler Polypeptide (VAP) on this mice model.

**Methods:**

We tested the effect of VAP on β-catenin conditional activation mice, and used Cre negative littermates as controls. Micro-CT, histology and histomorphometry analysis were performed to evaluate the curative effect of VAP on mice facet joint-like phenotype. Expression of β-catenin and collagen II was detected by immunohistochemistry (IHC) and western-blot., MMP13, ADAMTS4 and ADAMTS5 was detected by immunofluorescence (IF). RT-PCR analysis was preformed to detect mRNA expression of cartilage degrading enzymes, such as MMP13, ADAMTS4 and ADAMTS5.

**Results:**

Results of micro-CT (μCT) analysis showed that VAP could partially reverse lumbar disc osteophyte formation observed in *β-catenin(ex3)*^*Col2ER*^ mice. Histology data revealed VAP partially improved facet joint cartilage tissue invades. Histomorphometry analysis showed an increase in total cartilage area after VAP treatment. IHC show that VAP reduced β-catenin protein levels and moderately up-regulated collagen II protein levels. RT-PCR and IF data showed that VAP down-regulated the expression of extracellular matrix synthesis (ECM) degradation enzymes MMP13, ADAMTS4 and ADAMTS5.

**Conclusion:**

Taken together, VAP may modulate ECM by inhibits MMP13, ADAMTS4 and ADAMTS5 via Wnt /β-catenin signaling pathway. Velvet Antler Polypeptide may be a potential medicine for FJ-OA.

## Background

Facet-Joint Osteoarthritis (FJ-OA) is a common degenerative disease of the lumbar spine accounting for 15–45% of lower back pain (LBP) in 10–15% of young adults with chronic LBP and up to 40% of the older population with pre-existing trauma [1, 2].The spinal unit consists of intervertebral disc and the facet joint (FJ) [3]. Different from intervertebral disc, facet joints are the only synovial joints in the spine. Because of its special anatomical structure and location, facet joint play an important role in lumbar spine mobility regulating upper and lower vertebral body activity range, as well as maintaining intervertebral disc stability [2]. Because of their involvement in rotational kinematics, mobility, and force distribution in the lumbar spine, FJs are susceptible to degenerative changes and also considered as a main cause of low back pain and disability [1, 4, 5] Current treatment is limited to surgical intervention and analgesic drugs all of which cannot halt dieases progression. Many studies suggested that Wnt/β-catenin signaling pathway plays a critical role in osteoarthritis [6–8]. Di et al. [9, 10] observed that β-catenin levels were increased in human OA samples, and β-catenin conditional activation (cAct) mice which specifically over-express β-catenin gene in articular chondrocytes showed osteoarthritis-like phenotype and severe defects in intervertebral disc tissue. Janine et al. [11] report that, in FJ-OA patients the number of beta-catenin-positive chondrocytes in facet joint was significantly increased. In this study we observe that the mice show FJ-OA like phenotype, therefore combined with previous research we use this genetically modified mouse as our FJ-OA animal model.

Velvet antler (*Cervi Cornu Pantorichum*) is deer immature-ossification horn with thick hair. According to “Compendium of materia medica”, Velvet antler has been used in traditional Chinese medicine for invigorating the kidney, nourishing bone and prolonging life according. Velvet Antler Polypeptide(VAP)is a active compounds extracted from Velvet Antler (VA). Nowadays VAP is widely used to enhance sexual functioning, inhibit inflammation, promote chondrocyte proliferation and delay the aging process [12–16]. However, the research about the effects of VAP on facet joint osteoarthritis and its biochemical mechanism remains limited. VAP treated chondrocytes exhibits a reduction in metalloproteinases secretion, a balanced cartilage matrix metabolism Furthermore VAP treated chondrocytes exhibits a reduction in the proportion of early apoptotic cells suggesting VAP could play a role of the apoptotic pathway in osteoarthritic chondrocytes [17]. Based on the following data, we hypothesize VAP may partially rescue β-catenin conditional activation mice FJ-OA-like phenotype by the ECM. In this study, we were able to show VAP partially rescue of β-catenin conditional activation mice facet joint osteoarthritis-like phenotype and relieve the pain related behavior. VAP acts by modulating ECM through the inhibition of MMP13, ADAMTS4 and ADAMTS5 via Wnt /β-catenin signaling pathway and this may be the biochemical mechanism.

## Materials and methods

### Animals

Mice were generously provided by Pro. Di Chen (Rush university medical center, Chicago, IL,USA). In order to activated β-catenin gene in articular chondrocytes, *β-catenin*^*(ex3)Col2ER*^ mice were generated by breeding *β-catenin*^*(ex3)flox/flox*^ mice with *Col2-CreER*^*T2*^ transgenic mice [18, 19]. *β-catenin*^*(ex3)Col2ER*^ mice were originally observed by Pro. Chen [9, 10] they find out this mice model exhibits osteoarthritis-like phenotype in the lumbar disc and knee joint. After tamoxifen (TM) induction, β-catenin gene is overexpress in chondrocyte specific cell in this transgenic mice model. The research was approved by the ethics committee of Liaoning University of Traditional Chinese Medicine experimental animal ethics committee (Shenyang, China). All the mice were housed in a climate-controlled environment (22 ± 1 °C) and had free access to food and water. Mice were sacrificed by overdose anesthesia with sodium pentobarbital (100 mg/kg, IP). Fifty mice were divided into 5 groups of 10 mice per group, mixed sex are used in different groups. Groups included VAP different dosage treatment groups, OA model group and a control group. Mice were injected with TM (1 mg/10 g body weight/day, IP, daily for 5 consecutive days) at 2 weeks old. Control group is WT mouse (cre negative littermates), other 4 groups are cre positive mice with different treatment. After 2 month of normal feeding, 3 groups of cre positive mice were administered different concentrations of VAP (20 mg/kg, 40 mg/kg and 80 mg/kg body weight, IP) daily for 4 weeks, while OA model group and control group were injected with saline (Table 1)

**Table 1:** Mice group name, strain and treatment

Group Name	Mice Strain	Treatment
Control group	Cre- littermates	normal saline 4 weeks(i.p.)
Model group	*β-catenin*^*(ex3)Col2ER*^	normal saline 4 weeks(i.p.)
VAP low dose group	*β-catenin*^*(ex3)Col2ER*^	20 mg/kg VAP 4 weeks(i.p.)
VAP medium dose group	*β-catenin*^*(ex3)Col2ER*^	40 mg/kg VAP 4 weeks(i.p.)
VAP high dose group	*β-catenin*^*(ex3)Col2ER*^	80 mg/kg VAP 4 weeks(i.p.)

### Drug

The drug used in this study was the Velvet Antler Polypeptide which was identified and extracted from the Red deer antler (*Cervus elaphus Linnaeus*) by Pro. Feng Li (School of Pharmacy, Liaoning University of TCM, Shenyang, China) (Data Unpublished).First, we carefully removed the villi of the antler, sawing antler into pieces and crushing them into grains. Water was used as the solvent to combine ultrasonic extraction with ethanol precipitation. To maximize VAP extraction and purification, the L9 (3^4^) orthogonal design experiment was carried out by using Bradford protein assay to determine the content of valvet antler polypeptides as the evaluation index. According to the orthogonal test result, the water extraction optimum technological conditions is as follows, size (80–100 mesh), solid-liquid ratio(1:12), extract 3 times, 20 min each time. We then used the Kay nitrogen analyzer to detect the content of protein polypeptide on antler powder and water residue after extraction. This step was to further verify the result of Bradford protein assay. After water extraction we used alcohol precipitation technology to extract VAP, the relative concentration of the liquid was 0.5 g/ml (crude drug), concentration of ethanol was 65%, precipitate time was 4 h. Tricine-SDS-PAGE gel electrophoresis analysis showed 9 higher resolution bars. Then we used different MFL-B membrane(PP-100、PS-50、HPS-10、HPS-5、HPS-3) to separate and purify VAP at different concentrated solutions weight different molecular weight. The highest content VAP fragment under 10 kDa is 3-5 kDa (20.6%), therefore we chose VAP (3-5 kDa) as our experiment drug. At last, 3–5 kDa VAP concentrated solution was placed in - 80 °C Ultra-low temperature refrigerator (DW86W100, Haier, China) for 24 h, and then put in lyophilizer ALPHA 1-4D_plus_(CHRIST, German) for 46 h to get lyophilized powder. With the pre-column derivatization method, the contents of amino acids in the velvet antler peptide segment was determined by amino acid analyzer and ODS column. The column temperature was set at 27 °C, wavelength was 360 nm and flow rate was 1.2 mL/ min. The results indicate that the correlation coefficients of amino acids in velvet antler peptide segment were all greater than 0.997.The precision, stability and repeatability of RSD were less than 3% and the recovery was all between 96.6–104.7% with RSD less than 2.4%. The content of binding amino acid in 3-5 kDa VAP is 266.3 mg/g, the content of binding essential amino acid in 3-5 kDa VAP is 39.5 mg/g.

### Micro-computed tomography (μCT)

Spine tissues from 5 groups were subjected to analysis of the changes in bone structure using a μCT 80 Specimen micro-computed tomography scanner (Scanco Medical, Brüttisellen, Switzerland) with a 55 kVp source and a 72 μAmp current. We scanned the lumbar spine at a resolution of 10 μm. The scan images from each group were evaluated at the same thresholds to allow 3-dimensional structural rendering of each sample.

### Histology and Histomorphometry

After μCT analysis, part of disc tissues was frozen with liquid nitrogen immediately for western-blot and RT-PCR analysis. Part were fixed in 10% NB-formalin for 3 days, decalcified in 14% EDTA for 14 days, and then embedded in paraffin. Several facet joint sections (3 μm thick) were cut for histology and IHC analysis. Histology test was stained with Alcian Blue Hematoxylin/Orange G (ABH/OG).

Histomorphometry measurements were performed using OsteoMeasure software (OsteoMetrics, Inc. Atlanta, GA, USA). Alcian blue-stained articular cartilage areas were outlined on projected images of each histology section to show facet joint’s articular cartilage area.

### Immunohistochemistry (IHC) and immunofluorescence (IF) analysis

IHC analysis was performed using non-phospho (active) β-catenin (Ser45) (1:800 dilution; CAT#:19807, Cell signaling technology, USA), collagen II antibody (1:200 dilution; CAT#: ab34712, abcam, Britain).

IF analysis detected MMP13 antibody (1:300 dilution; CAT#: ab39012, abcam, Britain), Admts4 (1:400 dilution; CAT#:ABT178, Millipore, Billerica, MA), and Adamts5 antibody (1:500 dilution; CAT#: ab41037, abcam, Britain).

### Western-blot

Lumbar disc tissues were ground in liquid nitrogen. Total protein was extracted using RIPA buffer (Beyotime, Biotechnology). Sample was separated in 10% SDS-PAGE and transferred to a PVDF membrane then subjected to immunoblotting with non-phospho (active) β-catenin antibody (1:200 dilution) overnight. HRP conjugated secondary antibodies (1:10000 dilution; Vazyme, Biotech) for 1 h and the protein existence was detected by ECL (Beyotime, Biotechnology).

### Total RNA extraction and real-time RT-PCR analysis

Lumber disc tissues were harvested to extract total RNA using Trizol (Invitrogen, Carlsbad, CA, USA) according to the manufacturer’s protocol. Total RNA was used to synthesize cDNA by GoTaq® 1-Step RT-qPCR System (CAT#:A6001, Promega, USA). Primer names and sequences for real-time PCR are listed in Table 2.

**Table 2:** RT-PCR primer sequence

Gene	Sequence
GAPDH F	GACATGCCGCCTGGAGAAAC
GAPDH R	AGCCCAGGATGCCCTTTAGT
mmp13 F	GCAGCTCCAAAGGCTACAA
mmp13 R	CATCATCTGGGAGCATGAAA
Adamts4 F	GGCAGATGACAAGATGGCAGCATT
Adamts4 R	AGACGAGTCACCACCAAGTTGACA
Adamts5 F	CCAAATGCACTTCAGCCACGATCA
Adamts5 R	AATGTCAAGTTGCACTGCTGGGTG

### Statistical analysis

Data were regression analysis with SPSS13.0 by one-way ANOVA. Results were considered significantly different at *P* < 0.05 or *P* < 0.01. Values are expressed as mean ± standard deviation (SD).

## Result

### Effect of VAP on *β-catenin*(*ex3*)^*Col2CreER*^ mice facet joint-like phenotype

**Fig. 1:**
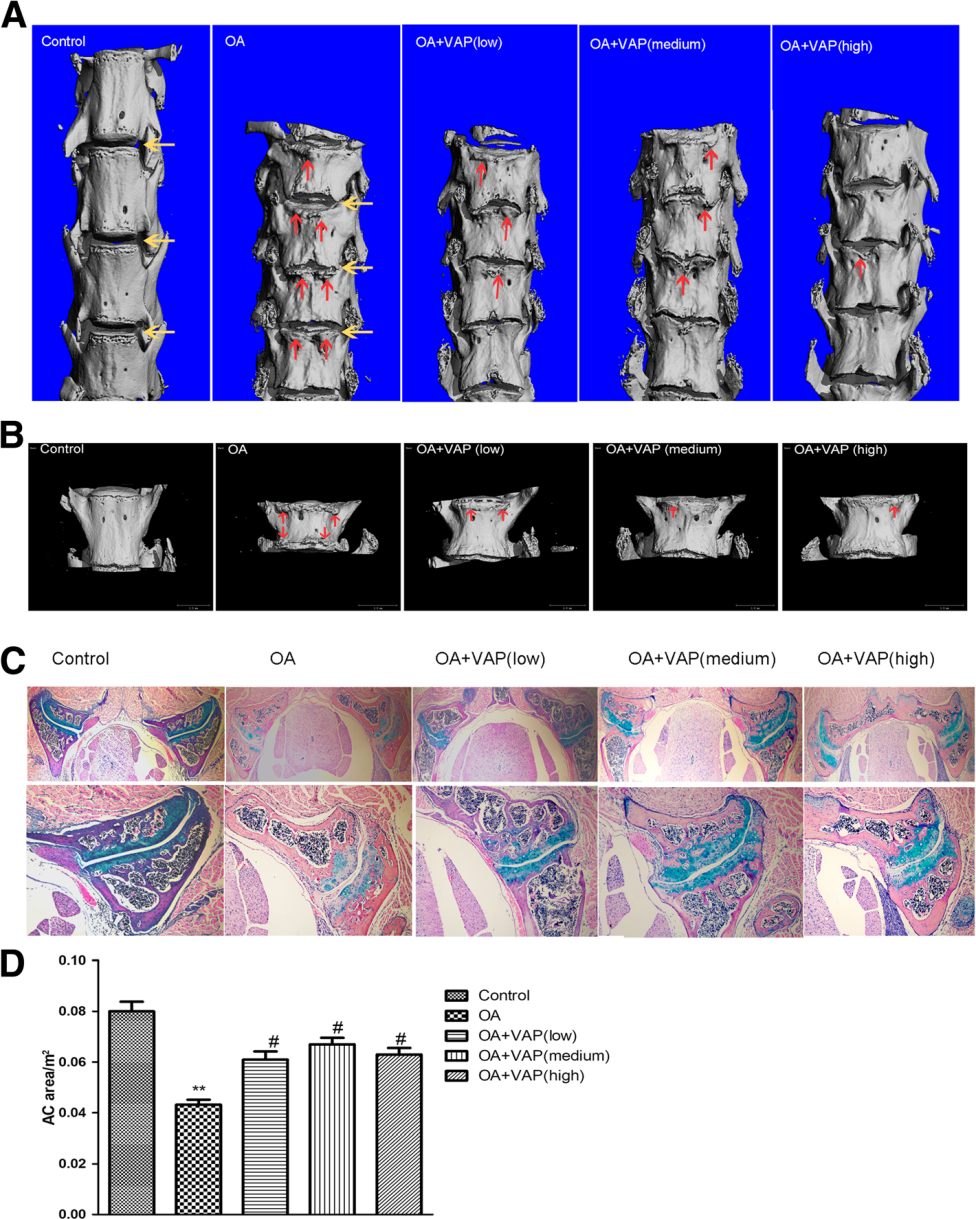
VAP partially rescue β-catenin(ex3)^Col2CreER^ mice facet joint-like phenotype. OA model group showed osteophytes formation (red arrow) and disc space narrowing (yellow arrow) on L1-L4 (**a**). However, the osteophyte formation around the lumbar intervertebral disc decreased in VAP different dosage groups, especially in 4th lumbar vertebral (**b**). Micro-CT (μCT) data show VAP partially rescue β-catenin(ex3)^Col2CreER^ mice lumbar disc osteophyte formation. Histologic results demonstrated that facet joint phenotype of the WT group is normomorph, the articular cartilage surface is smooth and complete, cartilage layer is clear, chondrocyte under the articular cartilage arranged orderly. Histology of model group displayed severe loss of facet joint cartilage tissue compare with wild type group, part of the cartilage surface appear damage, cartilage layer dye and thinning. After treat with VAP, the number of facet joint growth plate chondrocytes increase. Representative photos show the morphology using microscopy (**c**). Histomorphometry analyze the articular cartilage area of each histology section. Alcian blue-stained areas were outlined on projected images to determine facet joint’s articular cartilage area. * *P* < 0.05,** *P* < 0.01 vs Control group; ^#^*P* < 0.05,^##^*P* < 0.01 vs OA group. Data were expressed as mean ± SD. The results indicate VAP may increase FJ articular cartilage area (**d**)

Micro-CT data showed that compared with the control group, the mice of OA model group showed osteophytes formation and disc space narrowing on lumbar spine (Fig. 1a). However, the osteophyte formation around the lumbar intervertebral disc decreased in VAP different dosage groups, especially in 4th lumbar intervertebral disc (Fig. 1b). The data indicate that VAP may partially improve lumbar spine osteophyte formation.

Results of ABH/OG staining showed that facet joint morphology of the control group is normomorph, the articular cartilage surface is smooth and complete, cartilage layer is clear, chondrocytes under the articular cartilage arranged orderly. In the OA model group, facet joint were disorganized, part of the cartilage surface appear damage, cartilage layer dye and thinning, chondrocytes arranged and local crack produces, cavity appear in cartilage lacuna. Histologic results demonstrated facet joint osteoarthritis phenotype, including severe loss of cartilage, a reduced number of growth plate chondrocytes, and subchondral bone showed hyperplasia and hypertrophy (Fig. 1c).

The histomorphometry analysis the alcin blue-stained areas were outlined on projected images of each histology section to determine facet joint’s articular cartilage area showed that compared with the control group, the articular cartilage area of OA model group significant decrease (*P* < 0.01). After treat with VAP, the articular cartilage area of mice facet joint dramatic inreased (*P* < 0.05). The results indicate VAP may increase FJ articular cartilage area (Fig. 1d).

### VAP modulate ECM synthesis by inhibits MMP13, ADAMTS4 and ADAMTS5 via Wnt /β-catenin signaling pathway

**Fig. 2:**
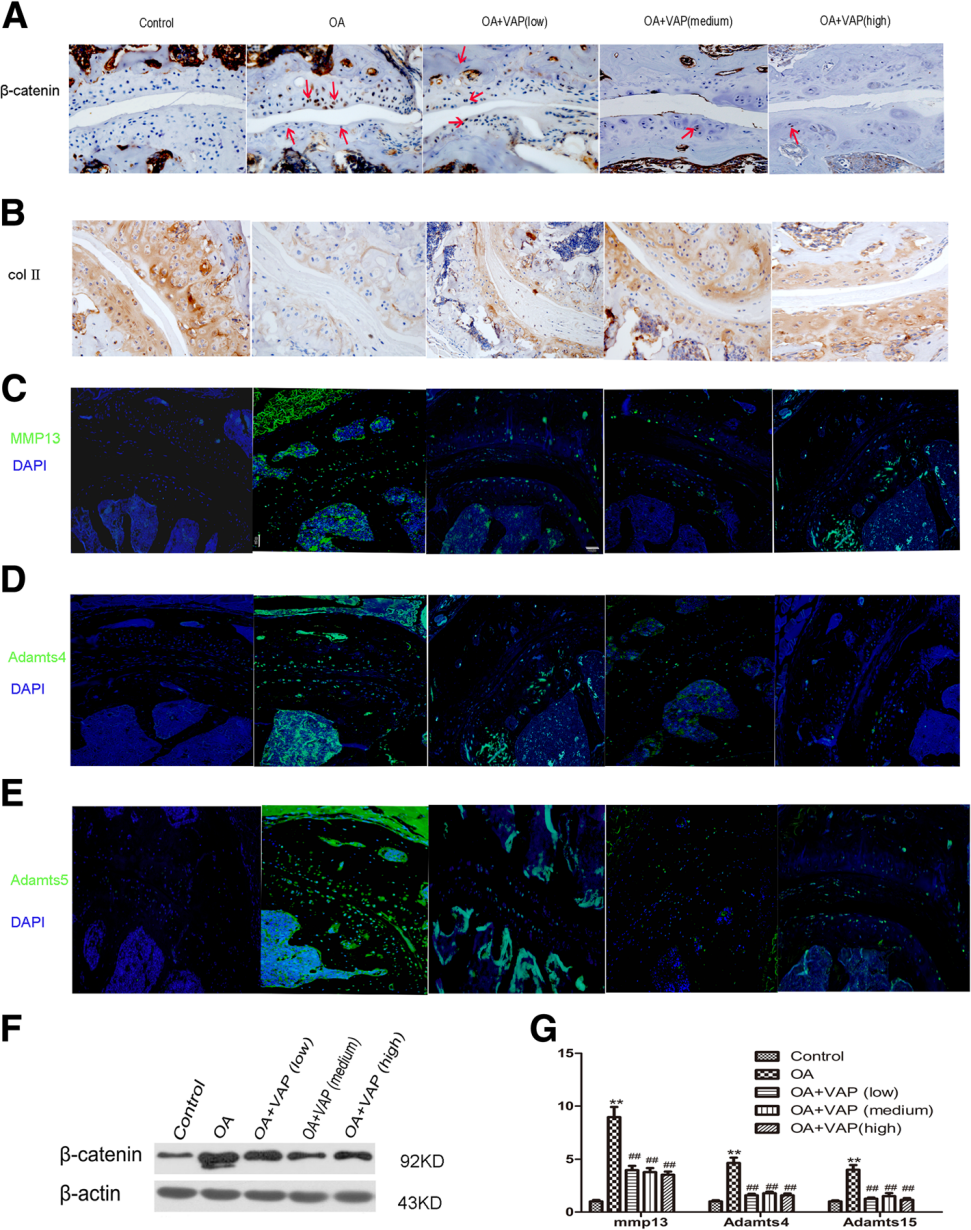
VAP modulate ECM synthesis by inhibits MMP13, ADAMTS4 and ADAMTS5 via Wnt /β-catenin signaling pathway. Immunohistochemistry of β-catenin (**a**) and collagen II (**b**) were staining in mice facet joint disc. β-catenin was express in chondrcyte (dark brown) and collagen II immunoreactivity were evident throughout the entire cartilage matrix (light brown). IHC analysis showed that VAP regulate the expression of β-catenin and collagen II protein level in facet joint. IF showed MMP13 (**c**), Admts4 (**d**) and Admts5 (**e**) protein levels were significantly increased in OA model group compare with control group. In VAP groups, MMP13, Admts4 and Admts5 protein levels were reduced. VAP regulate the gene expression encoding for ECM degradation in facet joint. Lumbar disc tissues were harvest to proceed wstern blot analysis (*n* = 5), the result indicate that compared with the WT group, protein level of β-catenin rise apparently in model group. Compared with model group, protein level of β-catenin declined in VAP different dosage group. VAP may partially downregulate the expression of β-catenin protein level (**f**). The mRNA expression of ECM degradation enzymes MMP13, ADAMTS4 and ADAMTS5 was evaluated by real-time polymerase chain reaction (*n* = 5). Values were expressed as mean ± SD. * *P* < 0.05,** *P* < 0.01 vs Control group; ^#^*P* < 0.05,^##^*P* < 0.01 vs OA group (**g**)

Expression of β-catenin and collagen II detected by IHC. β-catenin protein level was significantly increased but collagen II protein levels decreased in OA model group compare with control group, indicating that the chondrocytes underwent hypertrophy and lead to ECM degradation at this stage. In VAP groups, β-catenin protein levels reduced and collagen II protein levels moderately increased (Fig. 2a, b).

Gene expression encoding for ECM degradation was detected by IF. MMP13, Admts4 and Admts5 protein levels were significantly increased in OA model group compare with control group. In VAP groups, MMP13, Admts4 and Admts5 protein levels were reduced (Fig. 2c-e). Western blot result demonstrated that compared with the control group, protein level of β-catenin rise apparently in OA model group. VAP may partially down-regulate β-catenin protein level (Fig. 2f).

RT-PCR results indicated that VAP down regulated mRNA expression of Wnt /β-catenin Signaling pathway downstream target genes, such as MMP-13, ADAMTS-4 and ADAMTS-5. Compare with control group, MMP-13, ADAMTS-4 and ADAMTS-5 mRNA expression was raised in OA model group, *P* < 0.05, data was statistically significant. In VAP different dosage group, MMP-13, ADAMTS-4 and ADAMTS-5 mRNA expression decrease moderately, *P* < 0.05, data was statistically significant,but there was no significant difference between three different dosages of treatment groups (Fig. 2g).

## Discussion

Deer antler is a precious traditional Chinese medicine. According to compendium of materia medica, deer antler is deer unossified juvenile horn, with a rate of 1, 2 cm daily rapid growt. It can complete the whole process from bone cell differentiation development to mature in just 90 days, making it an effective and readily available treatment for invigorating the kidney, nourishing the blood, bones and bone morrow. Some researches indicate the VAP has an effect on the apoptosis of chondrocytes and may inhibit reduction of glycosaminoglycan and type II collagen in cartilage matrix. But the animal study remains scarce due to lack of mice model.

In the former study we examined β-catenin expression in patients with disc degeneration and we found that β-catenin protein was up-regulated in most disc samples, especially in the area with chondrocyte cluster formation. Furthermore, we have demonstrated that OA and IVD degeneration can occur through activation of Wnt/β-catenin signaling. β-catenin is the critical modulator in Wnt/β-catenin signaling pathway. In our study, we showed VAP cause continuous β-catenin degradation which prevents β-catenin accumulation and translocation to the nucleus. But how VAP inhibit β-catenin expression needs further exploration, therefore in vitro experiment should be perform to mimic the in vitro experimental.

Facet joints are the only synovial joint at the spine that contains in intervertebral joint. Two facet joints of the posterior column and one endplate-disk endplate joint of the anterior column formed intervertebral joint as a three-joint complex. Because of the high level of mobility and the large forces influencing lumbar FJ [2], FJ-OA is one of the main cause of low back pain and can result in immobility in the patients. The intervertebral disk and the facet joints interactively degenerate, causing altered stresses on the integrity and mechanical properties of the spinal ligaments, which results in degeneration of the spinal unit as a whole [20–22].

Articular cartilage contains chondrocytes and ECM secreted by chondrocytes. ECM mainly consist of collagen(mostly are typy II callagen) and proteoglycans (aggrecan is the main form). Collagen II, IX and XI connected to form a web, then proteoglycan fill in the web gap and bind to a large amount of water, these all work together to ensure the structural integrity and elasticity of articular cartilage. Aggrecanases and collagenase are key enzymes related to the degradation of ECM [23]. ADAMTS (a disintegrin and metalloproteinase with thrombospondin motifs), are extracellular, multidomain enzyme [24], among the family members, ADAMTS4 (aggrecanases-1) and ADAMTS5(aggrecanases-2) make the most contribution to aggrecan degradation [25–27]Besides ADAMTSs,,matrix metalloproteinases (MMPs) is another key enzyme, the process of collagen cleavage is mainly regulated by MMPs [28]. MMP13 is one of the most important MMPs, it cleaves fibrillar collagens with preference to type II collagen over type I and III collagens, and displays stronger gelatinase activity than other MMPs [29, 30] In this case, we displayed that VAP could partially modulate ECM synthesis by down regulate MMP-13, ADAMTS-4 and ADAMTS-5 mRNA expression, thus rescuing FJ-OA like phenotype suggesting VAP might be a potential medicine for FJ-OA .

## Conclusion

Taken together, VAP may modulate ECM by inhibits MMP13, ADAMTS4 and ADAMTS5 via Wnt /β-catenin signaling pathway. Velvet Antler Polypeptide may be a potential medicine for FJ-OA. Both inflammatory factors and nerve growth factors should be test to explore the mechanism of VAP release mice pain related behavior in our further study.

## Data Availability

The datasets used and/or analysed during the current study are available from the corresponding author on reasonable request.

## Abbreviations

ADAMTS: A disintegrin and metalloproteinase with thrombospondin motifs
ECM: Extracellular matrix synthesis
FJ-OA: Facet joint osteoarthritis
MMPs: Matrix metalloproteinases
VAP: Velvet Antler Polypeptide
